# The Value of Paratracheal Lymphadenectomy in Esophagectomy for Adenocarcinoma of the Esophagus or Gastroesophageal Junction: A Systematic Review of the Literature

**DOI:** 10.1245/s10434-021-10810-8

**Published:** 2021-11-29

**Authors:** Amaia Gantxegi, B. Feike Kingma, Jelle P. Ruurda, Grard A. P. Nieuwenhuijzen, Misha D. P. Luyer, Richard van Hillegersberg

**Affiliations:** 1 Department of Surgery, Vall d’Hebron Hospital Universitari, Barcelona, Spain; 2 Department of Surgery, University Medical Center Utrecht, Utrecht, The Netherlands; 3 Department of Surgery, Catharina Hospital Eindhoven, Eindhoven, The Netherlands

**Keywords:** Esophageal adenocarcinoma, Upper mediastinal lymphadenectomy, Nodal metastases

## Abstract

**Background:**

The role of upper mediastinal lymphadenectomy for distal esophageal or gastroesophageal junction (GEJ) adenocarcinomas remains a matter of debate. This systematic review aims to provide a comprehensive overview of evidence on the incidence of nodal metastases in the upper mediastinum following transthoracic esophagectomy for distal esophageal or GEJ adenocarcinoma.

**Methods:**

A literature search was performed using Medline, Embase and Cochrane databases up to November 2020 to include studies on patients who underwent transthoracic esophagectomy with upper mediastinal lymphadenectomy for distal esophageal and/or GEJ adenocarcinoma. The primary endpoint was the incidence of metastatic nodes in the upper mediastinum based on pathological examination. Secondary endpoints were the definition of upper mediastinal lymphadenectomy, recurrent laryngeal nerve (RLN) palsy rate and survival.

**Results:**

A total of 17 studies were included and the sample sizes ranged from 10-634 patients. Overall, the median incidence of upper mediastinal lymph node metastases was 10.0% (IQR 4.7-16.7). The incidences of upper mediastinal lymph node metastases were 8.3% in the 7 studies that included patients undergoing primary resection (IQR 2.0-16.6), 4,4% in the 1 study that provided neoadjuvant therapy to the full cohort, and 10.6% in the 9 studies that included patients undergoing esophagectomy either with or without neoadjuvant therapy (IQR 8.9-15.8%). Data on survival and RLN palsy rates were scarce and inconclusive.

**Conclusions:**

The incidence of upper mediastinal lymph node metastases in distal esophageal adenocarcinoma is up to 10%. Morbidity should be weighed against potential impact on survival.

Esophagectomy in the context of bi- or trimodality treatment achieves a 5-year survival rate of 40–50% in patients with locally advanced esophageal cancer.^1,2^ An adequate lymphadenectomy is an essential part of esophagectomy and most surgeons routinely remove at least the abdominal and mediastinal lymph node stations (i.e. two-field lymphadenectomy).^3^ Recent studies emphasized the need for a proper lymph node dissection in esophagectomy, as the lymph node yield is directly related to overall survival.^4,5^ Nonetheless, there is an ongoing debate regarding the mediastinal lymph node stations that should be dissected routinely. Particular disagreement exists on the role of an upper mediastinal lymphadenectomy involving the paratracheal lymph nodes (stations 2 and 4 or 105 and 106 according to the American and Japanese classification systems, respectively).^6,7^ While removal of these stations might improve locoregional control of disease, the paratracheal lymph nodes are located in a complex anatomical area containing the recurrent laryngeal nerves and several major vascular structures.^8–10^ A paratracheal lymph node dissection, particularly on the left side, is surgically challenging, especially in the open approach. Hence, the potential oncological merits of paratracheal lymphadenectomy need to be weighed against its possible risks in terms of surgical morbidity.

While upper mediastinal lymph node metastases most often occur with squamous cell carcinoma, several studies have shown that paratracheal lymph node metastases are also found in patients with distal adenocarcinoma, even after neoadjuvant therapy.^11–13^ In this perspective, the entire mediastinal peri-esophageal lymph node network should theoretically be removed to optimize the chances of curation and survival following esophagectomy for both adeno- and squamous cell carcinoma. However, available evidence on this topic is conflicting and there is substantial variation regarding the definition of an upper mediastinal lymphadenectomy.^14^ Moreover, there is controversy regarding the survival benefit of extensive mediastinal lymphadenectomy in patients who received neoadjuvant chemoradiation (nCRT).

To provide an objective overview of current evidence on the clinical value of paratracheal lymphadenectomy for adenocarcinoma of the distal esophagus or gastroesophageal junction (GEJ), a systematic review of the literature was performed.

## Methods

### Search Strategy

A systematic electronic literature search was performed using MEDLINE (via PubMed), EMBASE and the Cochrane Library. The search terms ‘resection’, ‘esophagectomy’, ‘esophageal resection’, ‘oesophagectomy’, ‘oesophageal resection’, ‘mediastinal lymph*’, ‘mediastinal node*’, ‘paratracheal’, ‘upper mediastin*’, ‘high mediastin*’, ‘upper chest’, ‘higher chest’, ‘station 2’, ‘station 4’, ‘2L’, ‘2R’, ‘4R’, ‘4L’, ‘station 105’, ‘106recR’ , ‘106recL’, ‘adenocarcinoma’, ‘distal esophageal tumor*’, ‘distal esophageal carcinoma’, ‘esophagogastric junction’, ‘esophago-gastric junction’, ‘gastroesophageal junction’ and ‘gastro-esophageal junction’ were used in combination with the Boolean operators ‘AND’ or ‘OR’. A full description of the search strategy is presented in Table 1. Two independent researchers (AG and FK) independently performed the electronic search in December 2020.

**Table 1:** Search strategy

Search terms
*MEDLINE/Pubmed*
1. (‘resection’ OR ‘esophagectomy’ OR ‘esophageal resection’ OR ‘oesophagectomy’ OR ‘oesophageal resection’)
2. (‘mediastinal lymph*’ OR ‘mediastinal node*’ OR ‘paratracheal’ OR ‘upper mediastin*’ OR ‘high mediastin*’ OR ‘upper chest’ OR ‘higher chest’ OR ‘station 2’ OR ‘station 4’ OR ‘2L’ OR ‘2R’ OR ‘4R’ OR ‘4L’ OR ‘station 105’ OR ‘106recR’ OR ‘106recL’)
3. (‘adenocarcinoma’ OR ‘distal esophageal tumor*’ OR ‘distal esophageal carcinoma’ OR ‘esophagogastric junction’ OR ‘esophago-gastric junction’ OR ‘gastroesophageal junction’ OR ‘gastro-esophageal junction’)
4. (‘lung’[title] OR ‘lung cancer’[tiab])
5. #1 AND #2 AND #3 NOT #4
*EMBASE*
(‘resection’:ti,ab,kw OR ‘esophagectomy’:ti,ab,kw OR ‘esophageal resection’:ti,ab,kw OR ‘oesophagectomy’:ti,ab,kw OR ‘oesophageal resection’:ti,ab,kw) AND (‘mediastinal lymph*’:ti,ab,kw OR ‘mediastinal node*’:ti,ab,kw OR ‘paratracheal’:ti,ab,kw OR ‘upper mediastin*’:ti,ab,kw OR ‘high mediastin*’:ti,ab,kw OR ‘upper chest’:ti,ab,kw OR ‘higher chest’:ti,ab,kw OR ‘station 2’:ti,ab,kw OR ‘station 4’:ti,ab,kw OR ‘2l’:ti,ab,kw OR ‘2r’:ti,ab,kw OR ‘4r’:ti,ab,kw OR ‘4l’:ti,ab,kw OR ‘station 105’:ti,ab,kw OR ‘106recr’:ti,ab,kw OR ‘106recl’:ti,ab,kw) AND (‘adenocarcinoma’:ti,ab,kw OR ‘distal esophageal tumor*’:ti,ab,kw OR ‘distal esophageal carcinoma’:ti,ab,kw OR ‘esophagogastric junction’:ti,ab,kw OR ‘esophago-gastric junction’:ti,ab,kw OR ‘gastroesophageal junction’:ti,ab,kw OR ‘gastro-esophageal junction’:ti,ab,kw) NOT (‘lung’:ab,ti OR ‘lung cancer’:ab,ti)
*Cochrane Library*
‘resection’ OR ‘esophagectomy’ OR ‘esophageal resection’ OR ‘oesophagectomy’ OR ‘oesophageal resection’ in Title Abstract Keyword AND ‘mediastinal lymph*’ OR ‘mediastinal node*’ OR ‘paratracheal’ OR ‘upper mediastin*’ OR ‘high mediastin*’ OR ‘upper chest’ OR ‘higher chest’ OR ‘station 2’ OR ‘station 4’ OR ‘2L’ OR ‘2R’ OR ‘4R’ OR ‘4L’ OR ‘station 105’ OR ‘106recR’ OR ‘106recL’ in Title Abstract Keyword AND ‘adenocarcinoma’ OR ‘distal esophageal tumor*’ OR ‘distal esophageal carcinoma’ OR ‘esophagogastric junction’ OR ‘esophago-gastric junction’ OR ‘gastroesophageal junction’ OR ‘gastro-esophageal junction’ in Title Abstract Keyword NOT ‘lung’ OR ‘lung cancer’ in Title Abstract Keyword (word variations have been searched)

### Study Selection, Eligibility Criteria

Titles and abstracts were scrutinized by the same two independent researchers (AG and FK) to determine their suitability for inclusion. The full text of potentially relevant articles was retrieved and assessed for inclusion by the same two authors. All studies reporting on the primary endpoint, i.e. the incidence of pathologically confirmed upper mediastinal lymph node metastases in patients undergoing transthoracic esophagectomy for distal esophageal or GEJ adenocarcinoma, were included. Secondary endpoints included the exact definition of performed upper mediastinal lymphadenectomy, recurrent laryngeal nerve injury rate, and survival. Upper mediastinal lymphadenectomy was defined as part of a total mediastinal lymphadenectomy.^13^ Case reports, studies with fewer than 10 patients, reviews, poster abstracts, animal studies, and non-English-language articles were excluded. If authors from the same institution had published a primary paper and then an updated analysis with a larger patient cohort, the most recent publication was included. The reference list of articles obtained was searched to identify additional articles. Any discordances between the two authors regarding study inclusion were resolved between all co-authors. The quality of all selected articles was scored according to the Centre for Evidence-Based Medicine (CEBM) Levels of Evidence, 2011 version.

### Data Abstraction

For eligible studies, data were extracted from the original articles, including publication year, country of origin, sample size, age, sex, histology, tumor location, neoadjuvant treatment, pathological T and N stage, definition of performed upper mediastinal lymphadenectomy, number of patients with pathologically positive nodes in the upper mediastinum, recurrent laryngeal nerve palsy rate, and survival. If data from any of the above items were not reported in the study, items were indicated as ‘NR’ (not reported). The extracted data were presented per study in tables. As the number of studies was limited and the variability in study design was considerable, no meta-analyses were performed. The Preferred Reporting Items for Systematic Reviews and Meta-Analyses (PRISMA) guidelines were followed for the conduction and reporting of this systematic review.

## Results

### Inclusion

**Fig. 1:**
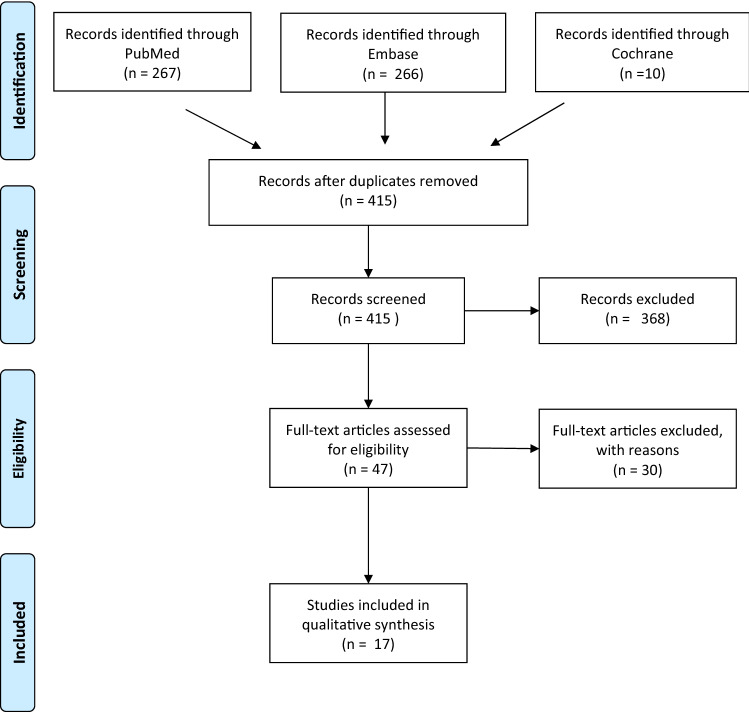
Flowchart systematic search

The primary literature search through the PubMed, Embase, and Cochrane libraries identified 543 studies. The search results and selection process are summarized in Fig. 1. The first screening discarded 496 papers, based on title and/or abstract, leaving 47 studies for full-text assessment. Thirty studies were excluded due to rates of upper mediastinal lymph node metastases not being reported (*n* = 19), mediastinal nodal status reported altogether without stratifying upper mediastinal lymph nodes (*n* = 5), reporting on fewer than 10 patients (*n* = 4), using the same case series as a more recent paper from the same authors (*n* = 1), and results not being reported in absolute numbers (*n* = 1). The remaining 17 studies^15–31^ were included in this review.

### Study Characteristics

The included studies were published between 2000 and 2020. Patient demographic factors as well as tumor and treatment characteristics are presented in Tables 2 and 3, respectively. Of the 17 included studies, 11 (65%)^17–20,23,24,26,28–31^ reported on Asian patient cohorts. Adenocarcinoma was the only histological subtype in 16 studies, and also represented 92% of the histological subtypes in one study.^20^ Furthermore, of the 17 included studies, 10 (59%)^15,19,20,22,24–27,29,30^ included patients who received neoadjuvant treatment in varying proportions (see Table 3); one study pretreated the full cohort,^15^ while the remaining studies applied neoadjuvant treatment to 11–59% of the cohort. As shown in Table 4, the sample sizes of the cohorts who underwent esophagectomy with upper mediastinal lymphadenectomy for adenocarcinoma ranged from 10 to 634 patients.

**Table 2:** Patient population and tumor characteristics in the 17 included studies

Reference	Year	Country	Sample size	Age, years	Sex		Histology		Tumor location				CEBM levels
					Male (%)	Female (%)	AC (%)	SCC (%)	Distal esophagus	GEJ			
										SI	SII	SIII	
Anderegg et al.^15^	2016	Netherlands	479	63.12	412 (86)	67 (14)	479 (100)	0	280	199			4
Dresner et al.^16^	2000	UK	104	62.9	91 (87.5)	13 (12.5)	104 (100)	0	0	104	0	0	4
Duan et al.^31^	2017	China	136	64	128 (94)	8 (6)	103	0	0	0	136	0	4
Han et al.^17^	2019	Korea	29	61.4	24 (82.7)	5 (17.2)	29 (100)	0	0	0	18	11	4
Kakeji et al.^18^	2012	Japan	129	NR	NR	NR	129 (100)	0	0	6	60	63	4
Kurokawa et al.^19^	2015	Japan	315	63	248 (78.7)	67 (21.2)	315 (100)	0	0	0	315	0	4
Kurokawa et al.^20^	2019	Japan	363	66	293 (80.7)	70 (19.2)	332 (91.5)	31 (8.5)	0	113	53	197	4
Lagarde et al.^21^	2005	Netherlands	50	61.5	45 (90)	5 (10)	50 (100)	0	0	0	50	0	4
Leers et al.^22^	2009	USA	509	64.4	Ratio	Ratio	509 (100)	0	301	208			4
Matsuda et al.^23^	2014	Japan	52	64	41 (78.8)	11 (21.1)	52 (100)	0	0	7	45	0	4
Minet et al.^24^	2019	Japan	69	56	65 (94.2)	4 (5.8)	69 (100)	0	0	69			4
Parry et al.^25^	2015	Netherlands	266	63	218 (82)	48 (18)	266 (100)	0	0	266			4
Sakaki et al.^26^	2020	Japan	44	63.4	41 (93)	3 (7)	44 (100)	0	0	19	25	0	4
Schröder et al.^27^	2002	Germany	51	60.9	49 (96)	2 (4)	51 (100)	0	0	51	0	0	4
Yamashita et al.^28^	2017	Japan	2384	68	1931 (81)	453 (19)	2384 (100)	0	0	86	1474	820	4
Yoshikawa et al.^29^	2016	Japan	381	63	305 (80)	76 (20)	381 (100)	0	0	381			4
Yura et al.^30^	2018	Japan	84	65.6	67 (80)	17 (20)	84 (100)	0	0	84			4

*AC* adenocarcinoma, *SCC* squamous cell carcinoma, *GEJ* gastroesophageal junction, *SI* Siewert I, *S2* Siewert II, *SIII* Siewert III, *CEBM* Center for Evidence-Based Medicine, *NR* not reported

**Table 3:** Treatment characteristics and staging

Reference	Sample size	Neoadjuvant treatment		(y)pTNM stage					
		Yes (%)	No (%)	T			N		
				T0–2	T3–4	Unknown	N0	N+	Unknown
Anderegg et al.^15^	479	CT: 293 (61) CRT: 186 (39)	0	193	283	3	226	253	0
Dresner et al.^16^	104	NR	NR	NR	NR	NR	NR	73	NR
Duan et al.^31^	136	0	136 (100)	17	118	0	44	92	0
Han et al.^17^	29	0	29 (100)	11	18	0	6	23	0
Kakeji et al.^18^	129	0	129 (100)	43	64	2	?		
Kurokawa et al.^19^	315	CT: 44 (14)	271 (86)	57	258	0	75	240	0
Kurokawa et al.^20^	363	121 (33.3)	242 (66.7)	124	234	5	111	247	5
Lagarde et al.^21^	50	0	50 (100)	7	43	0	4	46	0
Leers et al.^22^	509	94 (18.5)	415 (81.5)	NR	NR	NR	241	268	0
Matsuda et al.^23^	52	0	52 (100)	22	30	0	20	32	0
Minet et al.^24^	69	CT: 29 (42)	40 (58)	NR	NR	NR	NR	NR	NR
Parry et al.^25^	266	CT: 127 (48) RT: 1 CRT: 30 (11)	108 (41)	29	65	6 no malign	37	63	0
Sakaki et al.^26^	44	CT: 15 (34) CRT: 1	28 (64)	11	33	0	11	33	0
Schröder et al.^27^	51	CRT: 19 (37)	32 (63)	26	25	0	23	28	0
Yamashita et al.^28^	2384	0	2384 (100)	1861	523	0	1708	676	0
Yoshikawa et al.^29^	381	CT: 41 (11)	340 (89)	135	246	0	136	165	0
Yura et al.^30^	84	12 (14)	72 (86)	48	36	0	46	38	0

*CT* chemotherapy, *RT* radiotherapy, *CRT* chemoradiotherapy, *NR* not reported

**Table 4:** Sample size, definition and incidence of positive nodes in the upper mediastinum

Reference	*N*	Definition of the upper mediastinal lymphadenectomy performed	Patients with upper mediastinal lymphadenectomy performed	Number of patients with positive lymph node upper mediastinum	
			*N*	*N*	%
Anderegg et al.^15^	479	Paratracheal (st 2 and 4 R), aortapulmonary window (st 5) and subcarinal (st 7, 10r and 10l)	479	21/479	4.4
Dresner et al.^16^	104	Paratracheal	104	NR	5
Duan et al.^31^	136	Superior mediastinum	10	1/10	10
Han et al.^17^	29	Upper mediastinum	12	1/12	8.3
Kakeji et al.^18^	129	Upper mediastinal (105, 106)	129	NR	2
Kurokawa et al.^19^	315	Upper mediastinal	18	3/18	16.7
Kurokawa et al.^20^	363	Upper mediastinal (105, 106recL, 106recR)	67	6/67	8.9
Lagarde et al.^21^	50	Proximal lymph nodes: right paratracheal, aortopulmonary window and subcarinal	50	11/50	22
Leers et al.^22^	509	Paratracheal	250	3/250	1.2
Matsuda et al.^23^	52	105, 106recR, 106recL	52	4/52	16.6
Mine et al.^24^	69	Upper mediastinal (above tracheal bifurcation)	69	14/69	20
Parry et al.^25^	266	Upper mediastinal (paratracheal, aortapulmonary window, subcarinal)	111	NR	11
Sakaki et al.^26^	44	Upper mediastinal (105, 106recL, 106recR, 106tbL)	44	12/44	27.3
Schröder et al.^27^	51	Upper mediastinum	51	5/51	9.8
Yamashita et al.^28^	2384	Upper mediastinal (105, 106r, 106tb)	634	3/634	0.4
Yoshikawa et al.^29^	381	Upper mediastinal nodes	19	3/19	15.8
Yura et al.^30^	84	105 + 106	30	5/30	16.6

*NR* not reported

### Primary Outcomes

**Fig. 2:**
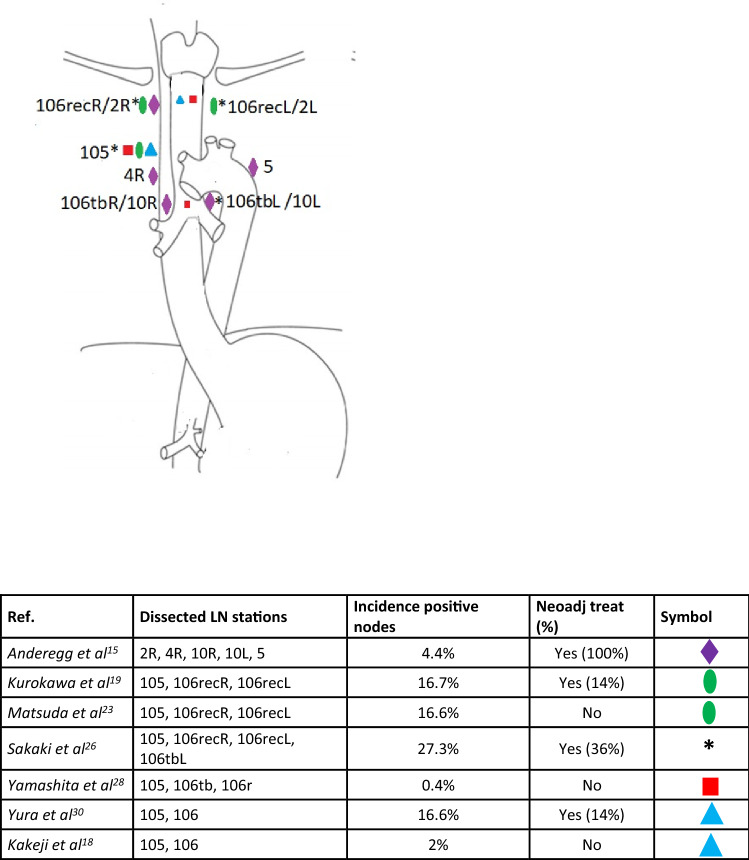
Anatomical representation and incidence of positive nodes as reported by 7 of the 17 included studies that clearly defined the performed upper mediastinal dissection. *LN* lymph node, *Neoadj treat* neoadjuvant treatment

There was no uniform definition for upper mediastinal lymphadenectomy among the studies, as demonstrated by Table 4. Of the 17 included studies, only 7 (41%)^15,18,20,23,26,28,30^ clearly specified which lymph node stations were dissected; one study^15^ followed the American Joint Committee on Cancer (AJCC) classification system to report the dissected lymph node stations, five studies^18,20,23,28,30^ followed the Japan Esophageal Society (JES) classification, and one study^26^ used both classifications (Fig. 2). In the remaining 10 studies,^16,17,19,21,22,24,25,27,29,31^ the exact lymph node stations that were dissected as part of an upper mediastinal dissection were not defined.

Among all 17 included studies, the median number of upper mediastinal lymph node metastases was 10.0% (interquartile range [IQR] 4.7–16.7). In 9 of the 17 studies (53%),^17,19,20,22,25,28–31^ patients were selected to undergo upper mediastinal lymphadenectomy based on preoperative diagnostics, and, in those patients, the median number of tumor-positive lymph nodes was 8.9% (IQR 1.2–11.0). In the cohorts that reported unselected series of patients undergoing upper mediastinal lymphadenectomy,^15,16,18,21,23,24,26,27^ the median number of tumor-positive lymph nodes was 9.8% (IQR 4.4–20.0).

In the seven studies that reported on patients undergoing a primary resection,^16–18,21,23,28,31^ the median number of tumor-positive nodes in the upper mediastinum was 8.3% (IQR 2.0–16.6). In one study that applied neoadjuvant therapy to the full cohort,^15^ the incidence of tumor-positive nodes in the upper mediastinum was 4.4%, while in nine studies that included patients treated with neoadjuvant treatment in varying proportions (11–59%),^19,20,22,24–27,29,30^ the median number of positive nodes in the proximal chest was 10.6% (IQR 8.9–15.8).

### Overall and Disease-Free Survival

Survival data for patients with pathologically positive nodes in the upper mediastinum after esophagectomy were reported in six studies, as shown in Table 5. Two studies reported on 5-year overall survival that ranged between 17 and 44.4%.^19,26^ One study^15^ reported a median disease-free survival of 15.4 months, while the remaining three studies reported on median survival (8 months),^21^ 5-year disease-free survival (0%),^24^ and 3-year overall survival of 53%.^30^

**Table 5:** Survival data from patients with pathologically positive nodes in the upper mediastinum after esophagectomy

Reference	5-year OS (%)	5-year DFS (%)	Median survival (months)	Median DFS (months)
Anderegg et al.^15^	NR	NR	NR	15.4
Kurokawa et al.^19^	17%	NR	NR	NR
Minet et al.^24^	NR	0%	NR	NR
Lagarde et al.^21^	NR	NR	N+ proximal chest: 8	NR
			N− proximal chest: 25	
Sakaki et al.^26^	41.7–44.4	NR	NR	NR
Yura et al.^30^	53.3 (3 years)	NR	NR	NR

*OS* overall survival, *DFS* disease-free survival, *NR* not reported

### Recurrent Laryngeal Nerve Palsy Rate

Only one study reported on the recurrent laryngeal nerve (RLN) palsy rate, which occurred in 8 of 129 patients in that study (6.2%).^20^ Note that this rate was from all patients submitted to transthoracic esophagectomy in that case series, and was not specific for patients who received an upper mediastinal lymphadenectomy (67 patients in that group).

## Discussion

Based on this systematic review, the mean incidence of upper mediastinal lymph node metastases was up to 10% in patients undergoing transthoracic esophagectomy for distal esophageal or GEJ adenocarcinoma. Substantial variation was found in the reported incidences, which is probably at least partially explained by different definitions being used for an upper mediastinal lymphadenectomy. Furthermore, in most studies (9/17), selected patients underwent mediastinal lymph node dissection.

Transthoracic esophagectomy is currently considered to achieve the best oncological outcomes, as it allows a thorough mediastinal lymphadenectomy to maximize the chances of removing all affected lymph nodes. Data regarding the optimal extent of lymphadenectomy for esophageal adenocarcinoma in the era of neoadjuvant treatment are contradictory. On the one hand, there is evidence in favor of extensive lymphadenectomy conferring survival benefit; a meta-analysis including 26 studies supported the benefit of an increased lymph node yield from esophagectomy for overall and disease- free survival, even after neoadjuvant treatment.^4^ A study in Asian subjects evaluated the impact of dissecting specific lymph node stations on survival and concluded that paratracheal lymphadenectomy has therapeutic value in patients with esophageal carcinoma;^12^ however, this study could not be included in the current systematic review since it mainly included patients with squamous cell carcinoma and did not separately report the outcomes of patients with adenocarcinoma. A significant overall survival benefit was not found in selected patients when comparing transhiatal resection with a limited lymphadenectomy versus transthoracic esophagectomy with an extended lymphadenectomy.^32^ A trend towards improved long-term survival at 5 years using the extended transthoracic approach compared with the limited transhiatal approach was found in another study.^33^ Nevertheless, data on dissecting specific lymph node stations are lacking and most surgeons do not routinely dissect the upper mediastinal lymph nodes for distal esophageal and GEJ tumors, as the balance between potential oncological merits and risk of recurrent laryngeal nerve injury is not clear. This systematic review found that the incidence of upper mediastinal lymph node metastasis is as high as 10% in these patients, suggesting that involvement of the upper mediastinal nodes may be present, even in distal adenocarcinomas.

Ideally, preoperative imaging should identify these lymph nodes, allowing surgeons to select patients who are most eligible for upper mediastinal lymphadenectomy. However, the reliability of clinical lymph node staging remains poor as the sensitivity of positron emission tomography/computed tomography (PET/CT) scanning for the detection of esophageal lymph node metastases is only 34–74%.^34^ Although the sensitivity of endoscopic ultrasound (EUS) is somewhat higher for lymph nodes adjacent to the esophagus (75–84%), this diagnostic modality has a considerably lower specificity (65–75%) and is less accurate in identifying abnormal lymph nodes located more distantly.^35,36^ Although a combination of PET/CT and EUS is used in some centers for preoperative lymph node staging, diagnostic accuracy is generally considered to be insufficient to perform a targeted lymph node dissection. This could be an argument for esophagectomy with routine removal of all mediastinal lymph nodes at risk, including those located in the upper mediastinum. On the other hand, increased understanding of metastatic patterns based on tumor characteristics might carry potential to guide decision making. In a nationwide Japanese study that was included in this systematic review, it was shown that lymph node metastases in JES stations 105, 106L, and 106R (which correspond to AJCC stations 2 and 4) occurred in 0%, 0%, and 3%, respectively, of patients with GEJ adenocarcinoma or squamous cell carcinoma that extended <4 cm in the esophagus.^20^ Such insights, which are expected to be amplified within the next years by initiatives such as the TIGER trial (NCT03222895),^37^ might shed light on indications for performing a less aggressive lymph node dissection in particular subgroups in the future.

Although upper mediastinal lymph node metastases are observed in up to 10% of patients with distal esophageal adenocarcinoma, the impact of upper mediastinal lymphadenectomy on survival remains unclear based on this systematic review. Hence, high-quality evidence on this topic is lacking and more research is needed to evaluate the clinical value of upper mediastinal lymphadenectomy. Even if future studies are able to identify an association between upper mediastinal lymphadenectomy and survival after esophagectomy for adenocarcinoma, investigators should be aware of the phenomenon referred to as stage migration, which may be the result of harvesting more lymph nodes.^38^ Future studies should thus strive to improve our understanding of the role of upper mediastinal lymphadenectomy on staging and survival in patients undergoing esophagectomy for adenocarcinoma.

With potential survival benefits on one side of the scale, the risk of complications should be weighed up against the other. As paratracheal stations 2 and 4 are adjacent to the RLNs, dissection of this region might increase the risk of iatrogenic RLN injury, with reported incidences in experienced centers ranging from 5 to 26%.^39–41^ RLN injury can result in hoarseness and difficulty swallowing, which reduces quality of life and increases the risk of aspiration pneumonia.^42^ Although paratracheal lymphadenectomy may be especially challenging after neoadjuvant chemoradiotherapy and requires a learning curve, studies by a Taiwanese group showed that it is safe in experienced hands.^39,43^ Therefore, surgeons should consider a thorough teaching program when starting to perform a full paratracheal lymphadenectomy as part of transthoracic esophagectomy. Moreover, the technical benefits of robotic surgery in the form of enhanced three-dimensional view and improved dexterity by Endo-Wrist technology might improve the safety of an upper mediastinal dissection. This hypothesis is currently being investigated in a randomized controlled trial by the same Taiwanese group, which compares the effectiveness and RLN palsy rates in robotic versus thoracoscopic esophagectomy with paratracheal lymphadenectomy.^44^

The strength of this study is its clinical relevance, as it represents the first systematic review that aims to provide insight into the value of upper mediastinal lymphadenectomy for patients undergoing esophagectomy for adenocarcinoma with and without neoadjuvant therapy. One of the limitations of the study is the heterogeneity in neoadjuvant treatment and patient selection. Another limitation seems to be the wide variation in definitions that were used for an upper mediastinal lymphadenectomy in the existing literature, which in fact highlights the low level of evidence on the topic at this moment. In the absence of studies that are suitable for pooling of data in order to generate a reasonable level of evidence, esophageal surgeons are currently operating mainly based on their personal convictions or those of their former teachers. To increase the transparency and comparability of studies on this topic, future studies should report the exact lymph node stations that are part of the lymphadenectomy. Furthermore, the authors suggest the dissection of stations 2 and 4 in both sides as the only definition of a full upper mediastinal lymphadenectomy as part of a total mediastinal lymphadenectomy. By increasing the number of comparable studies, it will be possible to establish evidence-based recommendations on this topic.

## Conclusion

This study demonstrates that the incidence of upper mediastinal lymph node metastases is up to 10% in patients undergoing transthoracic esophagectomy for distal esophageal or GEJ adenocarcinoma. The diagnostic accuracy of current imaging techniques is insufficient for the detection of upper mediastinal lymph node metastases, and the effect of neoadjuvant treatment on node positivity in this region is unclear. Although the impact on overall survival is not clear, if morbidity could be reduced, surgeons could consider standard upper mediastinal lymphadenectomy for distal esophageal adenocarcinoma to potentially improve the oncological outcome.
